# Supplementary material for: The interactome of intact mitochondria by cross-linking mass spectrometry provides evidence for coexisting respiratory supercomplexes
Source: Mol Cell Proteomics. 2017 Dec 8;17(2):216–32. doi: 10.1074/mcp.RA117.000470 (PMC5795388; doi:10.1074/mcp.RA117.000470)
Supplement: Supplemental Data [file supp_RA117.000470_133922_0_supp_23978_fzffwf.zip › spectra_annotation/mito_spectra_annotation/307-1-5-1-5-1.pdf]

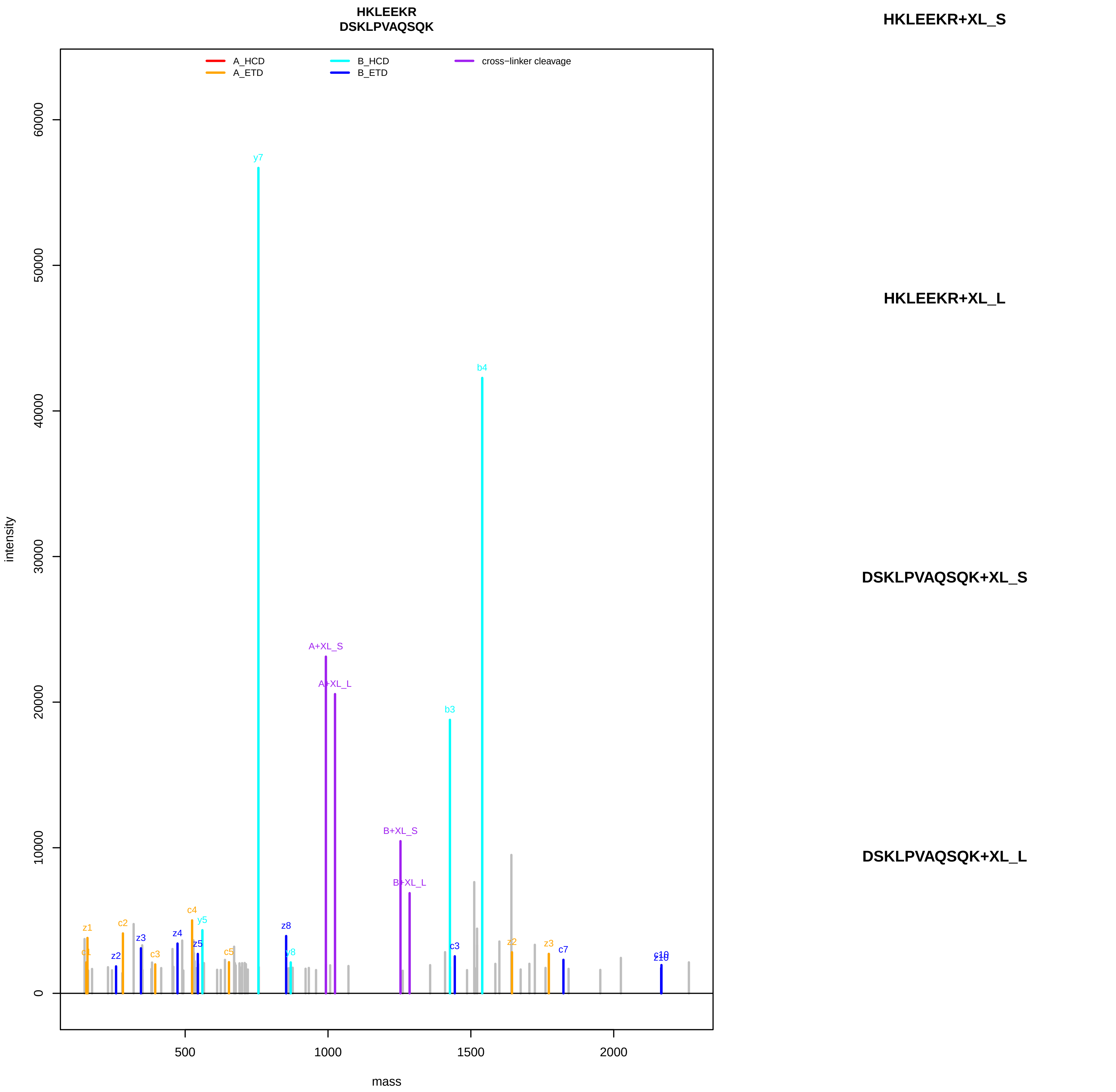
HKLEEKR
DSKLPVAQSQK
A_HCD
A_ETD
B_HCD
B_ETD
cross−linker cleavage
intensity
mass
0
10000
20000
30000
40000
50000
60000
500
1000
1500
2000
y7
b4
A+XL_S
A+XL_L
b3
B+XL_S
B+XL_L
z1
c1
c2
z2
z3
c3
z4
c4
y5
z5
c5
z8
y8
c3
z2
z3
c7
c10
z10
HKLEEKR+XL_S
HKLEEKR+XL_L
DSKLPVAQSQK+XL_S
DSKLPVAQSQK+XL_L
